# **Ob**sidian®ASG in **an**ast**o**motic healing after rectal cancer **res**ection—OBANORES: a prospective clinical feasibility study

**DOI:** 10.1007/s00384-025-04881-2

**Published:** 2025-04-05

**Authors:** Thomas Nikolas Valsamidis, Anders Tøttrup, Ken Ljungmann, Tue Højslev Avlund, Sanne Harsløf, Charlotte Buchard, Lene Hjerrild Iversen

**Affiliations:** 1 Department of Surgery, Aarhus University Hospital, Aarhus, Denmark; 2 Department of Clinical Medicine, Aarhus University, Aarhus, Denmark

**Keywords:** Anastomotic leakage, OBANORES, Colorectal cancer

## Abstract

**Purpose:**

Anastomotic leakage following rectal cancer resection is a serious complication. Despite efforts to prevent it, the risk remains high. Obsidian®ASG, an AUTOLOGOUS fibrin matrix with thrombocytes derived from the patient’s blood, shows promise but has not been thoroughly tested in rectal anastomosis. The aim of this study was to assess the feasibility of using Obsidian®ASG as a supplement in rectal anastomosis creation during minimally invasive rectal cancer resection.

**Methods:**

This prospective IDEAL stage 2a development cohort study included 50 patients undergoing rectal cancer resection with anastomosis using minimally invasive surgery at Aarhus University Hospital, Denmark. Obsidian®ASG application was assessed using a predefined rating scale: “Complete” (applied in all three prescribed steps), “Almost complete” (applied in at least the first or second step), and “Incomplete” (all others). Feasibility required “Complete” or “Almost complete” application in at least 90% of patients.

**Results:**

Obsidian®ASG application was “Complete” in 15 cases (30%) and “Almost complete” in 35 cases (70%), meeting feasibility criteria in all patients. No “Incomplete” applications occurred. Difficulties in achieving “Complete” application included anatomical constraints, material depletion, machine error, and time constraints.

**Conclusion:**

Obsidian®ASG was successfully applied in all patients undergoing minimally invasive rectal cancer surgery. These findings suggest its feasibility, but further large-scale, multi-center randomized trials are needed to fully assess its potential benefits for patient outcomes.

## Introduction

Colorectal cancer is one of the most frequent cancer diseases in Western countries, with more than 4000 new cases per year in Denmark [1]. Treatment with curative intent often includes surgery with a bowel resection. Here it is often possible to re-establish bowel continuity by creating an anastomosis. Unfortunately, the anastomosis does not always heal sufficiently, and an anastomotic leak (AL) may develop.

AL is a feared and serious complication, where bowel content will flow into the abdominal or pelvic cavity. Usually, AL occurs within the first 2 weeks after surgery [2]. The risk of AL following rectal cancer resection is 10–15%, whereas it is less than 3–4% following colon cancer resection [1]. Several factors appear to influence the risk of developing AL. They can be classified into preoperative factors such as patient- and disease-related characteristics, intraoperative factors such as surgical and anastomotic issues and surgeon’s experience, and perioperative factors such as use of medications that might impair healing. For rectal resections, the increased AL risk can, to some extent, be attributed to difficulties in performing the anastomosis in the narrow pelvic cavity; a challenging blood supply of the anastomosis, in close vicinity of anal sphincters; and male sex [3]. In the case of rectal cancer, having received neoadjuvant radio- chemo-therapy further increases the risk of AL [3]. To this day, there are no well-established, validated, internationally recognized prediction model for AL, and the incidence of AL is still unpredictable [3].

Several initiatives aiming to reduce the AL rate following rectal cancer resection have already been made. These include refinement of surgical and anastomotic techniques, intraoperative evaluation of blood supply of the oral bowel and anal rectal stumps, preoperative oral antibiotics and mechanical bowel preparation, and prohibition of non-steroidal inflammatory drugs and steroids in the perioperative course, among others [3, 4]. Despite these, the AL rate following rectal cancer surgery is still much too high.

In a pilot study, Shamiyeh et al. examined the benefit of applying an autologous fibrin matrix in combination with thrombocytes, named Obsidian®ASG, onto and around the anastomotic area, during colorectal anastomotic creation [5]. The rationale was that this would reinforce the anastomosis as well as strengthen the healing process. Obsidian®ASG was applied in all 271 cases included in this retrospective, two-center observational study. The AL rate was 2.3% (4/177) following colonic resection and 2.4% (2/84) following rectal resection. Although no comparison group was used, these AL rates are definitely promising, suggesting this method warrants further research.

Obsidian®ASG is prepared from the patient’s own blood, which following a processing leaves a fibrin matrix with a high concentration of embedded non-activated thrombocytes [5–7]. At the site of application, these thrombocytes are, over a period of 4–7 days, released and activated through natural proteolytic absorption and degradation of the fibrin matrix. Activated thrombocytes will release growth factors and hereby activate fibroblast differentiation and migration, promote angiogenesis, and stimulate wound healing [8]*.* The fibrin matrix of Obsidian®ASG acts therefore as a carrier material, which protects the growth factors and releases them slowly in the application area. However, it also acts as a hemostatic agent, due to its high elasticity and tensile strength, which leads to better tissue adherence [9]. These characteristics can be used to reinforce the anastomosis. Autologous fibrin sealant is increasing being evaluated in different clinical settings, like in pancreatectomy, to reduce the risk of pancreatic fistula [10], during pulmonary resection, to control alveolar air leak [11], during esophageal anastomosis, to reduce the risk of AL [12], during the treatment of anal fistulas [13], and others.

Due to the above reasons, and as part of multi-year process aiming to reduce the AL rate, our department planned to implement the use of autologous fibrin matrix in combination with thrombocytes, Obsidian®ASG, in restorative rectal cancer resection. However, first we aimed to assess the feasibility of successful use and application of Obsidian®ASG as a supplemental procedure in the creation of rectal anastomosis when performing minimally invasive rectal cancer resection.

## Methods

### Study design

This prospective cohort study was a single-center, observational, clinical study investigating the feasibility of applying Obsidian®ASG on rectal anastomosis during minimally invasive rectal cancer resection.

The study was approved by The Central Denmark Region Committees on Health Research Ethics (journal no. 1–10–72–187-20), registered at the Danish Data Protection Agency (1–16-02–60-22), preregistered at ClinicalTrials.gov (NCT05293054) [14] and conducted in accordance with the Strengthening the Reporting of Observational Studies in Epidemiology (STROBE) statement [15] and the IDEAL stage 2a framework [16].

Written informed consent was obtained from included patients and the Helsinki declaration was followed in all aspects.

### Setting

The study was conducted at Department of Surgery, Aarhus University Hospital (AUH) from December 2021 through November 2023. A follow-up of two years is planned for long-term outcome (mortality).

AUH is one of two primary referral centers managing all rectal cancer surgery in Central Denmark Region, with a population of approximately 1.3 mill inhabitants. Annually, approximately 40–50 patients underwent minimally invasive restorative rectal cancer resection [17]. Danish health care is free and tax-supported.

### Participants and eligibility

All patients with newly diagnosed rectal cancer are evaluated at a multidisciplinary team (MDT) conference as per standard clinical practice at our department. This evaluation was also used as screening for eligibility to this study.

The following inclusion criteria were used: presence of primary rectal cancer (adenocarcinoma) with the lower boarder within 15 cm from the anal verge assessed by rigid proctoscopy. Clinical UICC stage I-III at time of rectal cancer diagnosis. Patients had to have an age of at least 18 years, an ECOG performance status 0–2, and be deemed suitable for intended curative rectal cancer resection at MDT either by total mesorectal excision (TME) or partial mesorectal excision (PME). They also had to be scheduled for elective, minimally invasive surgery. Written and orally informed consent had to be acquired.

The following exclusion criteria were used: presence of distant metastatic disease or locally advanced rectal cancer requiring extended resection or benign lesions of the rectum or of another malignant disease within previous 2 years or inflammatory bowel disease; use of open surgery; inability and unwillingness to give informed consent; and pregnant (positive pregnancy test) or breast-feeding women.

Eligible patients were recruited when they visited the outpatient clinic, to receive information about minimally invasive resection with anastomosis (TME or PME).

### Intervention

Rectal cancer resection was performed using minimally invasive technique, specifically robot- assisted surgery, according to the standard operating procedure at AUH [18]. This also includes preoperative mechanical bowel preparation.

A total of six colorectal surgeons performed the intervention. Prior to the start of the study, the sponsor (LHI) and a colorectal surgeon had an on-site visit at Kepler University Clinic, Linz, Austria, to observe the use Obsidian®ASG by Professor A. Shamiyeh (author of the pilot study [5]. A product manager instructed theater nurses and all colorectal surgeons in the preparation of the autologous fibrin matrix by use of Vivostat® System (Vivostat A/S, Lillerod, Denmark). All colorectal surgeons were trained in the application of Obsidian®ASG with minimally invasive technique in the form of e-learning (instructional videos). Further, a meeting with Professor A. Shamiyeh was held shorty after commencing the study, the aim of which was to answer any of the surgeons’ questions that arose after the first few applications of Obsidian®ASG.

#### Preparation of the autologous fibrin matrix

When the patient was anaesthetized, 120 mL venous blood was collected for preparation of the autologous fibrin matrix. The blood was mixed with 300 mg tranexamic acid and transferred to the Vivostat® System processing unit. The mixture was heated, underwent centrifugation, and was merged with 30 units of Batroxobin. This process separated the mixture into a concentrate of fibrin I and thrombocytes, and excess serum, the latter of which was led into a waste chamber. The concentrate was then mixed with 3.5 mL of pH4 sodium acetate buffer, and was transferred into an empty syringe. The 5–6 mL concentrate was thus ready for application, and the syringe was loaded into the Vivostat® System applicator unit. This whole process took approximately 30 min.

During application, the applicator unit simultaneously delivered the fibrin- and thrombocyte-concentrate together with a pH10 buffer solution, ensuring a neutral pH necessary for Obsidian®ASG.

#### Application of Obsidian®ASG

When the rectal cancer resection was completed and the specimen was extracted, Obsidian®ASG was applied using minimally invasive technique and an endoscopic kit device connected to the applicator unit.

The application process was divided into 3 steps:

Step 1: 1.5–2 mL Obsidian®ASG was applied onto the rectal stump, with the circular stapler device inserted transanally into the rectal stump. The circular stapler was then closed, but not yet fired.

Step 2: 2.5–3 mL Obsidian®ASG was applied 360° around the anastomosis, while taking care not to increase tension on the anal intestine end. The circular stapler was then fired and removed. The water–air-leak test was performed according to standard clinical practice.

Step 3: The remaining part of the Obsidian®ASG was then sealed 360° around the anastomosis.

#### Postoperative treatment

Patients were managed according to standard clinical practice at AUH. In summary, rectal cancer patients follow an enhanced recovery program and have routine blood tests, including C-reactive protein (CRP) measurements at postoperative day 2 and day 5, and when clinically indicated. In case of an uneventful recovery, patients with no defunctioning ileostomy were discharged within 3–5 days following surgery, whereas patients with an ileostomy were discharged within 5–7 days postoperatively. Radiological and/or endoscopic examination of the anastomosis was only performed in case of clinical or biochemical (significant CRP increase) suspicion of anastomotic leak.

Patients who developed an anastomotic leak were evaluated and treated according to national guidelines [19].

#### Concomitant care and interventions permitted or prohibited during the study

All standards of care interventions, according to daily practice at AUH, were permitted and expected to be performed. Of note, systemic corticosteroid (dexamethasone) was not allowed to be administered just before surgery in patients who were expected to receive a rectal anastomosis.

Other experimental interventions on the anastomosis or interventions, which may influence the healing of the anastomosis, were prohibited during the study.

### Outcomes

#### Primary endpoint

The primary endpoint was the rate of successful use and application of Obsidian®ASG as a supplemental procedure in the creation of rectal anastomosis with minimally invasive technique.

This was defined as the surgeon being able to reinforce the anastomosis with Obsidian®ASG as described in above. The applications were assessed using the following predefined rating assessment scale:

1. “Complete”: Obsidian®ASG is applied as described in the application section above. The amount measures in milliliters are estimates meant to guide the surgeons, and small deviations can still count as “Complete” application
2. “Almost complete”: Obsidian®ASG is applied only on the rectal stump (step 1), or only around the anastomosis before the circular stapler is fired (step 2), or only on two out of the three steps.
3. “Incomplete”: Any application not fulfilling “Complete” or “Almost complete”.

Use and application of Obsidian®ASG was considered successful, when it matched the criteria for “Complete” or “Almost complete.” Before the study starts, we decided that being able to deem the use of Obsidian®ASG as feasible, we required a successful application in 45 out of 50 (90%) patients.

Any deviations in the application of Obsidian®ASG were reported in the electronic case report form (eCRF). All operations were routinely video monitored, allowing for a blinded assessment by a third person within 30 days in case of doubt.

#### Secondary endpoints

The secondary endpoints were the following.

The surgeon’s self-assessment of the user-friendliness of using Obsidian®ASG; time spent creating a rectal anastomosis with application of Obsidian®ASG; duration of surgery; intraoperative blood loss; anastomotic leak rate; length of hospital stay; re-hospitalization within 30 days after surgery; morbidity within 30 days after surgery graded ≥ 2 severity according to the Clavien-Dindo classification; and 30-day and 2-year mortality.

The surgeon’s self-assessment of the user-friendliness of using Obsidian®ASG was rated in three grades: (1) easy, (2) difficult, but can be performed, and (3) very difficult.

Time spent creating the anastomosis with application of Obsidian®ASG was defined as time spent from inserting the circular stapler device in the rectal stump, just before the application starts, until the application around the circular anastomosis had been completed.

Duration of surgery was defined as the length of time between the start of the surgical procedure (introducing Verres canula) and the end of surgery (closure of skin incision).

Blood loss was defined as the total intraoperative blood loss in milliliters as recorded by the anesthesiologist or anesthesiologist nurse at the end of the procedure, not including the blood draw required for creating Obsidian®ASG.

Anastomotic leak was defined according to the definition as described by DCCG and is measured within 30 days after surgery [19].

Length of hospital stay was defined as: Number of days from the day of surgery to the day of discharge, including both days.

All re-hospitalizations in the Central Denmark Region within 30 days after surgery, in any department, were recorded. This included the reason for re-hospitalization.

Morbidity within 30 days after surgery focused on surgical and medical complications graded ≥ 2 in severity according to the Clavien-Dindo classification [20].

For safety reasons, mortality within 30 days was recorded, while mortality within 2 years after surgery is awaiting.

### Data

#### Data collection and management

The patient’s medical record was the source document.

Data was registered in an eCRF in REDCap. The data management system ensured compliance with applicable legislation and regulation on data handling and safety.

### Study size

In this IDEAL stage 2a development study [16], we selected a study size of 50 patients as we considered it large enough to give a sufficient assessment of the feasibility of the method according to the primary outcome.

#### Statistical methods

Data are presented by resection type (PME/TME) with categorical variables presented as numbers and percentages and were tested by tested with Chi-squared test or Fisher’s exact test as appropriate. Assessment categories of the Obsidian®ASG application (“Complete,” “Almost complete,” and “Incomplete”) were transferred into two categories (Successful, Not successful). Continuous data are presented as medians with range. Non-normal distributed data were tested with Mann–Whitney test. A *P*-value < 0.05 was considered statistically significant.

Statistical analysis was performed in SPSS Statistics 29.0 (IBM Corporation, New York, USA).

## Results

**Fig. 1:**
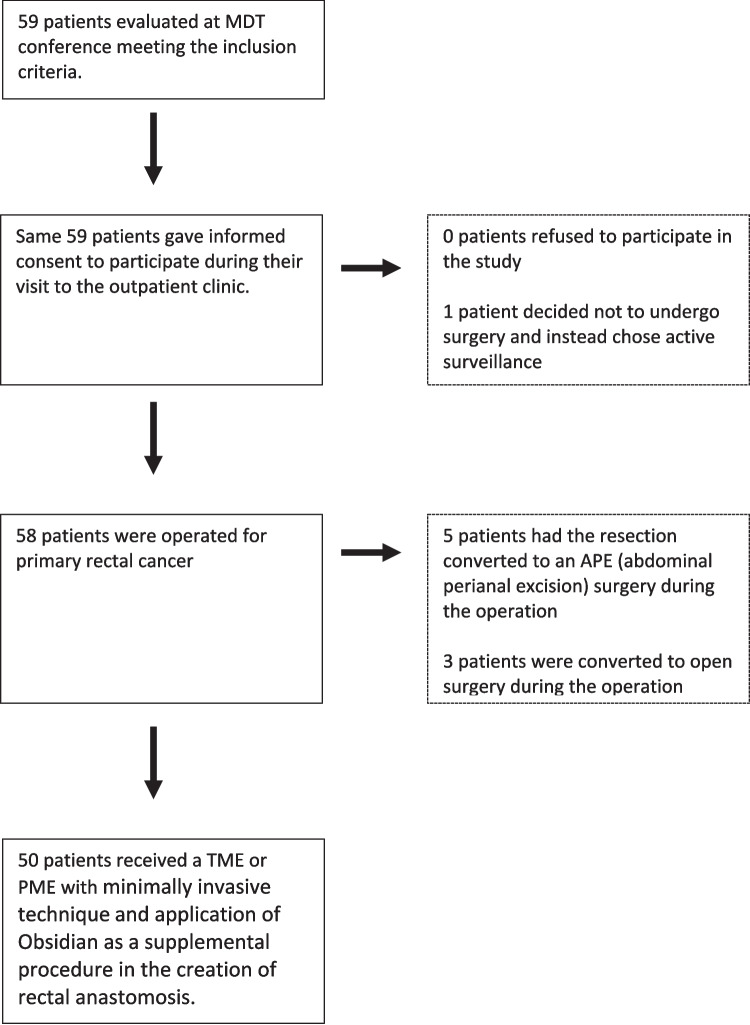
Patient inclusion

From December 2021 through November 2023, we included 50 patients undergoing minimally invasive rectal cancer resection with the application of the Obsidian®ASG matrix, Fig. 1. Of these, 17 (34%) received PME and 33 (66%) TME. A total of six surgeons performed the surgeries with a median of 9 (range 1–15) operations. Patient characteristics are summarized in Table 1. All tumors were adenocarcinomas.

**Table 1:** Patient characteristics

Variables		PME (*n* = 17)	TME (*n* = 33)	Total (*n* = 50)	*P-*value
Gender (male)		13 (76.5%)	22 (66.7%)	35 (70.0%)	0.474
Age		59 (50–74)	70 (44–78)	67 (44–78)	0.146
Body mass index (kg/m^2^)		26.5 (22.9–35.3)	25.4 (16.9–35.9)	25.7 (16.9–35.9)	0.084
Tobacco use	Current Previous Never	1 (5.9%) 8 (47.1%) 8 (47.1%)	6 (18.2%) 11 (33.3% 16 (48.5%)	7 (14.0%) 19 (38.0%) 24 (48.0%)	0.412
Alcohol consumption	0 1–13 ≥ 14	2 (11.8%) 14 (82.4%) 1 (5.9%)	9 (27.3%) 18 (54.5%) 6 (18.2%)	11 (22.0%) 32 (64.0%) 7 (14.0%)	0.150
Comorbidities	None Yes^a^	6 (35.3%) 11 (64.7%)	16 (48.4%) 17 (51.5%)	22 (44.0%) 28 (56.0%)	0.373
Performance status	0 1	17 (100%) 0 (0.0%)	32 (97.0%) 1 (3.0%)	49 (98.0%) 1 (2.0%)	1.000
Previous abdominal surgery	No Yes^b^	13 (76.5%) 4 (23.5%)	29 (87.9%) 4 (12.1%)	42 (84.0%) 8 (16.0%)	0.419
Tumor localization (cm) from the anal verge	11–15 6–10 0–5 cm	16 (94.1%) 1 (5.9%) 0 (0.0%)	5 (15.2%) 28 (84.8%) 0 (0.0%)	21 (42.0%) 29 (58.0%) 0 (0.0%)	< 0.001
cT category	1 2 3 4	1 (5.9%) 9 (52.9%) 6 (35.3%) 1 (5.9%)	2 (6.1%) 19 (57.6%) 12 (36.4%) 0 (0.0%)	3 (6.0%) 28 (56.0%) 18 (36.0%) 1 (2.0%)	0.390
cN category	0 1 2	13 (76.5%) 2 (11.8%) 2 (11.8%)	28 (84.8%) 5 (15.2%) 0 (0.0%)	41 (82.0%) 7 (14.0%) 2 (4.0%)	0.131
Preoperative radio-chemotherapy	Yes No	1 (5.9%) 16 (94.1%)	4 (12.1%) 29 (87.9%)	5 (10.0%) 45 (90.0%)	0.650
Postoperative diverting ileostomy	Yes No	0 (0%) 17 (100%)	32 (97.0%) 1 (3.0%)	32 (64%) 18 (36%)	< 0.001

Categorical variables are presented as numbers with percentages; continuous variables as medians with range.

^a^Comorbidities included heart disease (*n* = 22), diabetes mellitus (*n* = 7), lung disease (*n* = 2), and other comorbidities (e.g., various kinds of arthritis, aorta aneurysm) (*n* = 9), equally distributed according to type of resection. One patient may have one or more comorbidity.

^b^Previous abdominal surgery included appendicectomy (*n* = 6), cholecystectomy (*n* = 2), and hysterectomy (*n* = 2), equally distributed according to type of resection. One patient may have one or more previous abdominal surgery.

All 50 patients received an anastomosis with application of Obsidian®ASG according to our protocol and were included in the primary endpoint. However, three patients had intraoperative adverse events or developed postoperative complications excluding them from the analysis on anastomotic leakage and were excluded from all secondary endpoints. One of these had the anastomosis taken down immediately, as the oral bowel segment was twisted. Two patients also had their anastomosis taken down a few days after their operation, as the oral bowel segment was necrotic, owing to compromised blood supply. No defect on the anastomosis lining was detected during endoscopy and thus, not categorized as anastomotic leakage.

### Feasibility of Obsidian®ASG application

The Obsidian®ASG application according to the individual three steps are presented in Table 2. Step 1, i.e., application on the rectal stump while the circular stapler inserted, was performed in all patients. However, step 2, i.e., sealing 360° around the anastomosis before firing of the stapler, was only conducted in 38 (76%) patients. Step 3, i.e., sealing 360° around the anastomosis after firing and removal of the stapler, was only performed in 17 (34%) patients. The three application steps did not differ significantly among patients who underwent PME and TME.

**Table 2:** Rating of Obsidian®ASG applications

		PME (*n* = 17)	TME (*n* = 33)	Total (*n* = 50)	*P-*value
Application, individual steps	Step 1 Step 2 Step 3	17 (100.0%) 12 (70.6%) 5 (29.4%)	33 (100.0%) 26 (78.8%) 12 (36.4%)	50 (100.0%) 38 (76.0%) 17 (34.0%)	0.520 0.623
Application according to rating assessment scale	Complete^a^ Almost complete^b^ Incomplete^c^	5 (29.4%) 12 (70.6%) 0 (0.0%)	10 (30.3%) 23 (69.7%) 0 (0.0%)	15 (30.0%) 35 (70.0%) 0 (0.0%)	0.948
Combinations of steps in “Almost complete”^b^ application (*n* = 35)	Step 1 only Step 2 only Step 1 + 2 Step 1 + 3 Step 2 + 3	4 (33.3%) 0 (0.0%) 8 (66.7%) 0 (0.0%) 0 (0.0%)	5 (21.7%) 0 (0.0%) 16 (69.6%) 2 (8.7%) 0 (0.0%)	9 (25.7%) 0 (0.0%) 24 (68.6%) 2 (5.7%) 0 (0.0%)	0.481
User friendliness	Easy Difficult, but can be performed Very difficult	15 (88.2%) 2 (11.8%) 0 (0.0%)	19 (57.6%) 14 (42.4%) 0 (0.0%)	34 (68.0%) 16 (32.0%) 0 (0.0%)	0.028

Variables are presented as numbers with percentages.

^a^ “Complete”: Obsidian®ASG is applied as described in the application section above. The amount measures in milliliters are estimates meant to guide the surgeons.

^b^ “Almost complete”: Obsidian®ASG is applied only on the rectal stump (step 1), or only around the anastomosis before the circular stapler is fired (step 2), or only on two out of the three steps.

^c^ “Incomplete”: Any application not fulfilling “Complete” or “Almost complete”.

According to our predefined rating assessment scale, surgeons were able to reinforce the anastomosis successfully with Obsidian®ASG, either as “Complete” or “Almost complete,” in all 50 (100%) patients.

There were 15 “Complete”, 35 “Almost complete,” and 0 “Incomplete” applications (Table 2).

Among the 35 applications graded as “Almost complete”, the great majority (*n* = 24 (69%)) had step 1 and step 2 applications performed, whereas 9 (26%) patients only had step 1 performed and 2 (6%) had steps 1 and 3 performed.

Different reasons led to limited application of Obsidian®ASG resulting in an application rated as “Almost complete.” The main reason for failed application in step 2 was that, due to lack of space, it was not possible to get overview of the anastomosis, especially the posterior side, without the risk of pulling on the anastomosis (*n* = 11), and errors of application machine (*n* = 1).

Reasons, which precluded application in step 3 were challenges presented by anatomical constraints, such as a narrow pelvis or a general lack of sufficient space to visualize the posterior side of the anastomosis, especially in adipose men (*n* = 14); depletion of Obsidian®ASG (*n* = 13); and errors of application machine (*n* = 2). Additionally, in 4 instances, the operation dragged on, so the surgeon decided to skip the final step 3 due to time constraints.

The user friendliness of the application was evaluated through the aforementioned grading system. Thirty-four (68%) applications were categorized as “Easy”, 16 (32%) as “Difficult, but can be performed” and 0 (0%) as “Very difficult.” This data is also presented in Table 2.

### Intraoperative and postoperative outcomes

The average time spent on creating a rectal anastomosis supplemented with application of Obsidian®ASG had a median duration of 12 min (range 4–26) held against an overall duration of surgery of median 301 min (range 169–444) (Table 3). Intraoperative blood loss had a median of 100 mL (range 28–250 mL).

**Table 3:** Intraoperative outcomes and postoperative complications ≤ 30 days

	PME (*n* = 16)	TME (*n* = 31)	Total (*n* = 47)	*P–*value
Time spent creating a rectal anastomosis (min.)	10 (7–26)	13 (4–23)	12 (4–26)	0.550
Duration of surgery (min.)	257 (169–334)	319 (225–444)	301 (169–444)	< 0.001
Blood loss (mL)	100 (30–250)	100 (28–250)	100 (28–250)	0.558
Anastomotic leakage	1 (6.3%)	4 (12.9%)	5 (10.6%)	0.483
Length of hospital stay (days)	5 (3–11)	7 (4–41)	7 (3–41)	< 0.001
Rehospitalization	0 (0.0%)	6 (19.4%)	6 (12.8%)	0.060
Morbidity ≥ 2 according to the Clavien-Dindo classification	0 (0.0%)	14 (45.2%)	14 (29.8%)	0.001

Categorical variables are presented as numbers with percentages; continuous variables as medians with range.

The postoperative complications are shown in Table 3. AL occurred in 5 (10.6%) out of the 47 patients. These AL events were seen in 2 (14.3%) of the 14 patients for which the Obsidian®ASG application was graded as “Complete” and in 3 (9.1%) of 33 patients for which the application was rated as “Almost complete” (*P* = 0.627).

Six patients (13%) were rehospitalized, but for reasons not related to the use of Obsidian®ASG (dehydration (*n* = 2), hyponatremia (*n* = 1), high CRP levels (*n* = 1), small intestinal obstruction because of malfunctioning ileostomy (*n* = 1), and AL (diagnosed on postoperative day 13 four days after discharge *n* = 1)).

Morbidity graded ≥ 2 severity, excluding the AL, according to the Clavien-Dindo classification was seen in 14 (30%) of patients. These complications were exclusively seen in patients who underwent TME surgery and included conditions like high stoma output (*n* = 5), intestinal paralysis (*n* = 4), urinary retention (*n* = 4), pulmonary embolism (*n* = 2), dehydration (*n* = 2), small bowel perforation (*n* = 1), intraabdominal abscesses (*n* = 1), and COVID-19 infection (*n* = 1) and occurred as single events or in combination.

No mortality was observed within 30 days.

## Discussion

This IDEAL stage 2a development study investigated the feasibility of using an autologous fibrin matrix, Obsidian®ASG, as a supplemental procedure in minimally invasive rectal cancer resection with the potential to reduce AL. We found that the application of the Obsidian®ASG was successful based on a “Complete” or “Almost complete” application in all 50 surgical procedures. Further, in two-thirds of surgeries, the surgeons found the application in the minimally invasive setting “Easy” and in no surgeries did they assess it as “Very difficult.”

As 70% of the applications were indeed categorized as “Almost complete,” the argument can be made that the Obsidian®ASG matrix presents some issues in its application during minimally invasive rectal cancer resection. This doubt is further strengthened by the evaluation of its user friendliness, where surgeons found the application “Difficult, but can be performed” in one third of procedures. Application on the posterior part of the anastomosis was especially challenging for the surgeons, due to lack of sufficient space to visualize the anastomosis safely. Depletion of the Obsidian®ASG also contributed to the rating as “Almost complete.” Importantly, however, only one sixth of the entire patient cohort had exclusively a step 1 application.

In clinical practice, the implications of this study are promising. The use of the Obsidian®ASG matrix in rectal anastomosis has already been reported in a retrospective study of Shamiyeh et al. [5], who reported a low AL rate of 2.4% following rectal resection. Our study was not designed to investigate effect of the Obsidian®ASG matrix on anastomotic healing. We noted a somewhat higher AL rate of 10.6% although within the 95% CI of the national clinical quality indicator standard for rectal anastomotic leakage set at < 8% by the Danish Colorectal Cancer Group [17]. The observed AL rate was comparable to rates previously reported from our institution [18]. However, the discordant AL rates could stem from variations in surgical technique, patient selection, and use of neoadjuvant therapy, among others, underlining the necessity for standardized protocols in future trials. Here, we have shown that the application of Obsidian®ASG is feasible, and thus enabling implementation of this technique, should future research demonstrate a beneficial effect on the AL rate.

Some limitations should be mentioned. None of the rating assessment scale and the self-assessment scale were validated instruments. However, both were pre-defined before study start. Due to the nature of our study design, the definition of a feasible application was arbitrary. Although we strove to provide a fair definition, this can lead to questions about whether or not the application was indeed feasible. Moreover, even though we felt that an “Almost complete” application should be counted as feasible, as the most important steps in the process had been completed, others might criticize this. A further limitation is the single-center design, which may limit the generalizability of the findings. However, six different surgeons performed the 50 applications.

An important consideration in the use of Obsidian®ASG is the required 120 mL blood draw for its preparation and the iatrogenic blood loss this entails. While the Danish Colorectal Cancer Group (DCCG) guidelines ensure that colorectal cancer patients are routinely screened for anemia and treated with intravenous iron if needed [21], and while the overall intraoperative blood loss, seen in Table 3, was minimal due to the use of minimally invasive techniques, untreated anemia has been linked to decreased long term survival [22]. Interestingly, this same multicenter snapshot study found no association between anemia and postoperative complications or 30-day mortality.

Recent recommendations from an Italian multisociety consensus on patient blood management in major digestive surgery emphasize the importance of minimizing phlebotomy for laboratory blood tests when not clinically justified [23]. While the 120 mL blood loss is indeed small, and was not deemed problematic by The Central Denmark Region Committees on Health Research Ethics, the impact of preoperative blood draws should not be disregarded. In surgical settings with an anticipated high intraoperative blood loss or in patients with untreated anemia, it might be considered to avoid this blood loss, until the potential benefits of Obsidian®ASG are better understood.

For future research, our OBANORES trial lays the groundwork for larger-scale, multi-center studies to further investigate the efficacy of Obsidian®ASG. Randomized clinical studies with placebo-controlled use of Obsidian®ASG as the ongoing, international multi-center ORSY trial [24] or Obsidian®ASG versus other anastomotic reinforcement techniques will be essential to establish its effect on AL rates. It should also be investigated whether all three steps in the application are necessary to achieve the potential strengthened healing of a rectal anastomosis, or if certain steps can be omitted. Of notice, we found no difference in AL rate following “Complete” and “Almost complete” applications, recognizing our study was not powered to evaluate AL rate. Its impact on long-term outcomes, such as cancer recurrence rates and overall survival, as well as its effectiveness across diverse patient demographics should also be explored.

In conclusion, the OBANORES trial exhibits the successful integration of the Obsidian®ASG autologous fibrin matrix in minimally invasive rectal cancer resection. Given the nature of rectal cancer surgery and its complications such as anastomotic leakage, Obsidian®ASG could be instrumental in improving patient outcomes, though further research is mandated to validate these results, before this technique should be integrated into standard clinical practice.

## Data Availability

Data is provided within the manuscript or supplementary information files.
